# OP01 Impact Of Cost Heterogeneity On Assessing The Value Of Gene Therapies

**DOI:** 10.1017/S0266462324000655

**Published:** 2025-01-07

**Authors:** Antal Zemplenyi, Jim Leonard, Michael DiStefano, Kelly Anderson, Garth Wright, Nick Mendola, Kavita Nair, Robert Mcqueen

## Abstract

**Introduction:**

High-cost gene therapies strain the sustainability of healthcare budgets. Despite the potential long-term savings promised by certain gene therapies, realizing these savings faces challenges due to uncertainties regarding the treatment’s durability and a lesser-discussed factor: the true potential for cost offset. Our study aims to assess the cost-offset uncertainty for US Medicaid regarding recently approved gene therapies in hemophilia A and B.

**Methods:**

The analysis used 2018 to 2022 Colorado Department of Health Care Policy & Financing data to determine direct costs of standard of care (factor replacement therapy or emicizumab). Cost-simulation models over five- and ten-year time horizons estimated Colorado Medicaid costs if patients switched to gene therapy (valoctocogene roxaparvovec or etranacogene dezaparvovec) versus maintaining standard of care. Patients were included if aged 18 and over with ICD-10-CM codes D66 (hemophilia A) and D67 (hemophilia B). In the base case, severe hemophilia A was defined as requiring greater than or equal to six yearly factor VIII or emicizumab claims and moderate/severe hemophilia B requiring greater than or equal to four factor IX replacement therapy claims annually.

**Results:**

Annual standard-of-care costs were USD426,000 (SD USD353,000) for hemophilia A and USD546,000 (SD USD542,000) for hemophilia B. Valoctocogene roxaparvovec (hemophilia A) had incremental costs of USD880,000 at five years and −USD481,000 at 10 years. Sensitivity analysis revealed a 23 percent chance of break-even within five years and 48 percent within 10 years. Etranacogene dezaparvovec (hemophilia B) showed incremental costs of USD429,000 at five years and −USD2,490,000 at 10 years. Simulation indicated a 32 percent chance of break-even within five years and 59 percent within 10 years. Varying eligibility (≥4 to ≥15 standard-of-care claims) notably affected break-even; for example, valoctocogene roxaparvovec: 40 percent to 77 percent chance of break-even in 10 years.

**Conclusions:**

Our study highlights significant cost variation in the standard of care of patients eligible for gene therapies, adding to the uncertainty surrounding cost estimation and highlighting the importance of addressing this factor in risk-sharing agreements. The impact of varying eligibility criteria on cost offsets emphasizes the importance of carefully defining eligibility when using real-world data in the context of health technology assessment.

